# Neonatal Screening for Spinal Muscular Atrophy and Severe T- and B-Cell Lymphopenias in Andalusia: A Prospective Study

**DOI:** 10.3390/ijns11010011

**Published:** 2025-01-30

**Authors:** Beatriz De Felipe, Carmen Delgado-Pecellin, Mercedes Lopez-Lobato, Peter Olbrich, Pilar Blanco-Lobo, Josefina Marquez-Fernandez, Carmen Salamanca, Beatriz Mendoza, Rocio Castro-Serrano, Cristina Duque, Mariana Moreno-Prieto, Marcos Madruga-Garrido, Jose M. Lucena, Raquel M. Fernandez, Maria Ruiz-Camacho, Alberto Varona, Olaf Neth

**Affiliations:** 1 Pediatrics Infectious Diseases, Rheumatology and Immunology Unit, Institute of Biomedicine of Seville, University Hospital Vírgen del Rocío/CSIC/University of Seville, 41013 Seville, Spain; bdefelipe-ibis@us.es (B.D.F.); polbrich@us.es (P.O.); piblanlo@gmail.com (P.B.-L.); 2 Clinical Biochemistry Department, University Hospital Vírgen del Rocío, 41013 Seville, Spain; 3 Neuropediatric Service, University Hospital Vírgen del Rocío, 41013 Seville, Spain; 4 Department of Pharmacology, Pediatrics and Radiology, University of Seville, 41008 Seville, Spain; 5 Neonatology Unit, Virgen de Valme Hospital, 41018 Seville, Spain; mf.josefina@gmail.com; 6 Neonatology Unit, Hospital Universitario Virgen de Macarena, 41008 Seville, Spain; 7 Neonatology Service, Juan Ramón Jiménez Hospital, 21005 Huelva, Spainmachicuri@hotmail.com (M.R.-C.); 8 Neonatology Unit, University Hospital Vírgen del Rocío, 41013 Seville, Spain; 9 Hospital Viamed Santa Angela de la Cruz, Sevilla and Neurolinkia, 41018 Seville, Spainmapolgra@yahoo.es (M.M.-G.); 10 Unidad de Inmunología, University Hospital Vírgen del Rocío, 41013 Seville, Spain; 11 Department of Maternofetal Medicine, Genetics and Reproduction, Institute of Biomedicine of Seville, University Hospital Virgen del Rocío/CSIC/University of Seville, 41013 Seville, Spain; 12 Paediatrics Service of Riotinto Hospital, 21660 Huelva, Spain

**Keywords:** spinal muscular atrophy, SMA, SCID, newborn screening, TRECs, KRECs

## Abstract

Spinal muscular atrophy (SMA) and severe T- and/or B-cell lymphopenias (STBCL) in the form of severe combined immunodeficiencies (SCID) or X-linked agammaglobulinemia (XLA) are rare but potentially fatal pathologies. In January 2021, we initiated the first pilot study in Spain to evaluate the efficacy of a very early detection technique for SMA and SCID. RT–PCR was performed on prospectively collected dried blood spots (DBSs) from newborns in Western Andalusia (Spain). Internal and external controls (SCID, XLA and SMA) were included. The determination of SMA was relative (positive/negative) and that of TRECs and KRECs was quantitative (copies/punch). A total of 14.035 prospective samples were analysed. All controls were correctly identified while no cases of SMA or SCID/XLA were prospectively identified. DBS analysis of infants with suspected SMA or STBCL that presented to our centre showed pathological values in two cases each for SMA and SCID and one for XLA, all of them being subsequently confirmed genetically. In this prospective pilot study, no infants with SMA or STBCL were detected; however, the technique applied here was shown to be reliable and fast, further supporting the benefits and need to include SMA and SCID in national newborn screening (NBS) programs, as it will allow early supportive and curative therapy.

## 1. Introduction

Spinal muscular atrophy (SMA) and severe T- and/or B-cell lymphopenias (STBCL) are severe diseases associated with high morbidity and mortality. Early diagnosis significantly impacts the patient’s outcome. SMA is one of the most common neuromuscular disorders in childhood and the second most frequent genetic cause of infant mortality [1]. SMA is an autosomal recessive neurodegenerative disorder caused by deletions or mutations in the survival motor neuron 1 (*SMN1*) gene and has a reported incidence of approximately 1 in 6000-10,000 live births [2,3,4]. SMA causes the degeneration and loss of alpha motor neurons in the anterior horn of the spinal cord, which leads to progressive muscle weakness, respiratory failure and death in severe cases. Clinical expression will be influenced mainly by the number of copies of the *SMN2* gene, a homologous gene that produces only 10% of the functional SMN protein. Higher *SMN2* copy numbers in patients with SMA are associated with a later onset and milder disease [5,6]. 

SMA is classified into five subtypes, 0 to 4, based on age at onset and best functional status expected. Another approach is based on severity (severe, intermediate and mild) and on the ability to achieve important motor milestones (sitting, standing/walking) [7,8]. SMA type I is the most severe and common type, which accounts for about 50% of patients. Life expectancy is generally considered to be <2 years, due to the rapid progression of muscle weakness and respiratory failure [6,7,9]. The loss of motor neurons is progressive and irreversible and the success of novel treatments depends largely on early intervention [9,10]. Only a genetic test allows its identification before the onset of symptoms, thereby offering the chance for early treatment.

In recent years, successful treatment has been demonstrated with gene therapy, such as by replacing the *SMN1* gene or using antisense oligonucleotides to modify the splicing of SMN2 to include exon 7 [11,12,13]. Currently, there are three therapies for SMA type I, II and III approved by the FDA and the EMA, using different strategies to enhance functional full-length SMN protein levels: Nusinersen (an antisense oligonucleotide); Onasemnogene Abeparvovec-xioi (gene-replacement therapy); and Risdiplam (survival of motor neuron 2 (*SMN2*) splicing modifier) [14,15]. Clinical trials have demonstrated early treatment to be critical to modify disease progression whilst improving health outcomes and life expectancy for patients with SMA.

STBCL in the first days of life may indicate an inborn error of immunity (IEI) such as severe combined immunodeficiency (SCID) or X-linked agammaglobulinemia (XLA). The prevalence varies depending on the type of immunodeficiency, but both SCID and XLA are rare, affecting 1 in 40,000–58,000 and 1–8/1,000,000) [16,17]. Curative treatments are available for patients with SCID using hematopoietic stem cell transplantation (HSCT) or gene therapy [18,19,20] and are currently under investigation for patients with XLA [21,22]. Offering curative therapy for SCID patients is more than 90% effective if performed within the first three months of life and in the absence of infection [18,23].

Due to the severity of the above pathologies and the importance of early diagnosis, they have been included in newborn screening (NBS) programs in several countries [24,25]. NBS using real-time PCR (RT-PCR) to screen for severe T cell lymphopenia in newborns was initiated first in 2008 [16] and later, a pilot study demonstrated the feasibility of using this approach for NBS for SMA [26,27]. A recent pilot study demonstrated the combined implementation of NBS for SCID and SMA to be cost-efficient whilst improving the quality of life of children and families affected by these conditions [28]. The aim of this study was to perform a combined screening for SMA and STBCL in the population born in Western Andalusia (Spain). We here present data of the first prospective study in Spain including SMA and STBCL in NBS.

## 2. Methods

### 2.1. Study Design, Setting and Population

Prospective, observational and longitudinal study to determine *SMN1*, TRECs and KRECs levels in dried blood samples (DBSs) obtained from neonates born in hospitals in Andalusia, Spain, between January 2021 and March 2022. This study was approved by the local ethics committees. Samples were included once the legal guardians signed informed consent forms.

### 2.2. Sample Collection

The technique was performed using the same sample collected for routine heel prick testing. Heel prick blood samples were dropped on Schleicher & Schuell #912 filter paper 48 h postpartum as part of the routine neonatal screening process. Two 3.2 mm discs were punched for each sample and stored at 4 °C until use. Once routine screening was completed, TRECs/KRECs and *SMN1* determination was performed using the same sample after consent was obtained.

### 2.3. SMN1, TRECs and KRECs Determination

**Figure 1:**
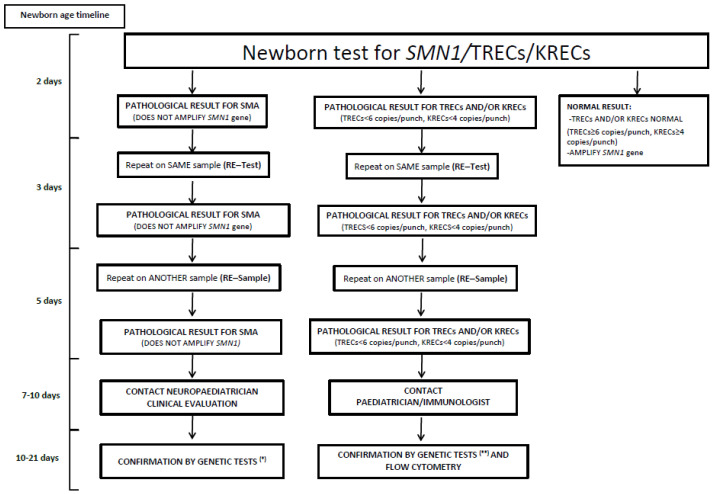
Algorithm for the interpretation of newborn *SMN1*/TRECs/KRECs testing. Absence of amplification of the *SMN1* gene suggests a positive result for SMA and samples with TRECs < 6 copies/punch and/or KRECs < 4 copies/punch are considered pathological results and suggest immunodeficiencies. In these cases, a re-test is required using the same dried blood sample. If the pathological result persists, the test is repeated on another dried blood sample (re-sample). If the pathological results continue, a specialist is contacted for a clinical evaluation and to perform specific tests. (* MLPA: Multiplex Ligation-dependent Probe Amplification; ** NGS: Next Generation Sequencing).

*SMN1*, TRECs and KRECs were evaluated by real time PCR (RT–PCR) using specific probes and primers (LightMix^®^KIT TREC KREC SMA Newborn, Roche-Tibmolbiol). Briefly, a 3.2 mm diameter disc of a DBS was deposited in a 96-well plate. After DNA elution and purification (DNA Elution and Purification Solution, Qiagen, Maryland, USA), real time PCR was performed using specific probes for the *SMN-1* (SMA), TREC and KREC genes (LightMix^®^ Kit TREC KREC SMA Newborn Screening, Roche Diagnostics, ref 40-0621-04) as previously described [29,30]. The SMA determination was qualitative (positive/negative), with the absence of amplification indicating the absence of the *SMN1* gene and, consequently, potential SMA. The TRECs/KRECs assay analysis was determined by absolute quantification as described above [30]. The following cutoff scores were defined for an estimated sensitivity of 99.8% for the detection of severe T-cell and/or B-cell lymphopenias: TRECs < 6/punch, KRECs < 4/punch [30]. The amplification cycle (Ct) of ACTB-actin beta indicates the efficiency of DNA extraction from the DBS samples (Ct ≥ 26). Figure 1 summarizes the algorithm of the sample processing and procedure in relation to the results obtained.

Different controls for SMA and primary immune deficiency diseases (PIDDs) were randomly included throughout the study. The internal controls were composed of DNA from patients from our centre diagnosed with SMA (5), IL7-R SCID (2), IL2Rγ-SCID (1) and XLA (1), while the external controls were provided by the NBS Quality Assurance Program of the Centers for Disease Control and Prevention (CDC), USA (10 SMA, 10 PIDDs).

Results interpretation is summarized in Figure 1. The determination of TRECs/KRECs and *SMN1* was performed on the same DBS used for routine screening in Western Andalusia. In cases of indeterminate or pathological results, the technique was repeated using the same DBS (re-test). If the result was repeatedly pathological or insufficient, a second DBS sample was requested (re-sample). The determination of the *SMN1* gene was qualitative (positive/negative). In the case of no amplification of the *SMN1* gene, a persisting negative result (after re-test and re-sample) suggested a homozygous deletion of exon 7 of the *SMN1* gene and was considered a suspected case of SMA. In this case, the neonate was referred to the neuropaediatric department for clinical evaluation and specific clinical tests were performed to corroborate the results. These tests, including genetic analyses using Multiplex Ligation-Dependent Probe Amplification (MLPA), were performed using a mixture of probes specific for the *SMA* locus in the same way as described above [31]. This technique confirmed the absence of the *SMN1* gene and also quantified the number of copies of the homologous *SMN2* gene and was developed by the local genetic department.

The analysis of the TRECs/KRECs assay was conducted as previously described [29,30]. Interpretation of the results is summarized in Figure 1. In cases of abnormal results (after the re-test and re-sample test), the newborn was clinically evaluated by the Paediatric Infectious Diseases and Immunology Unit and immunological studies, including the immunophenotype using flow cytometry as well as Next Generation Sequencing (NGS) with targeted analysis of 461 genes associated with Inborn Errors of Immunity (IEI) using the Twist HCExome_v2 Kit with the NextSeq 500 sequencing system platform (Illumina) at the Clinical Immunology laboratory of the Virgen del Rocio Hospital, Seville were performed [11].

This study was conducted under consideration of the current legislation, the ethical regulations of the Helsinki Declaration, and the guidelines on good clinical laboratory practice and was evaluated by the Ethical and Scientific committees of the participating centres. Data were recorded in accordance with the guidelines of the hospital ethical committee. For this study, written informed consent was obtained from a parent or legal guardian of the newborn.

### 2.4. Statistical Analyses

Quantitative variables were tested (Kolmogorov–Smirnov test or Shapiro–Wilk test) for a normal distribution and are expressed as mean ±standard deviation (SD) or as median and interquartile range (IQR) as appropriate. Qualitative variables are shown as absolute frequencies and percentages. Rates for abnormal, inconclusive, and normal results in the TRECs/KRECs-assay were calculated (95% CI). To assess the assay reliability, the proportion of false-positive results was calculated. For qualitative variables the chi-squared or the Fisher’s exact test was applied to estimate variable associations with T- and B-cell lymphopenias. For quantitative variables the Student’s *t* test for independent samples or the Mann–Whitney U test were performed. Correlations were assessed by means of the Pearson tests. *p*-values of <0.05 were considered as statistically significant. All statistical operations were performed using the IBM software SPSS Statistics version 26.

## 3. Results

From January 2021 to March 2022 a total of 14,035 newborns born in Western Andalusia were prospectively analysed. A total of 14,387 reactions were performed and 207 (1.47%) repetitions of the same DBS sample (re-test) were necessary due to TRECs or KRECs under the cutoff or no amplification of the *SMN1* gene and 145 (1.0%) were repeated due to insufficient material (re-sample) or persistent pathological results. Figure 1 shows the algorithm of the procedure in relation to the results obtained.

**Figure 2:**
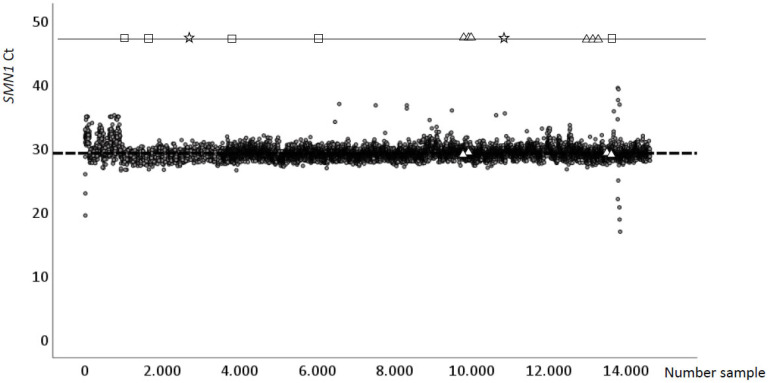
CT *SMN1* values from DBS and controls grey 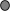 prospectively enrolled newborns; 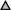 CDC controls; 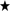 SMA suspected cases; 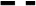 grey dotted line: Mean Ct *SMN1*; 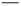 Detection limit (*Ct* = 45).

No cases of SMA were detected prospectively. Figure 2 shows the results of the 14,035 newborns screened prospectively for SMA. The mean cycle threshold (Ct) of the *SMN1* gene was 29 (±0.9). Only 21 (0.15%) repeat samples (re-test) were necessary due to pathological or indeterminate results, and the repeat was performed on the same DBS sample and a second sample (re-sample) was not necessary. Five internal and ten CDC controls were included, all of them being correctly detected (six of them non-amplifying and four amplifying for the *SMN1* gene). In addition, two newborn samples (47 and 28 days old; case A and B, respectively) were retrospectively included in the study after SMA suspicion. The DBSs were available in the metabolic laboratory (reference centre for the NBS of Occidental Andalusia). In both infants (cases A and B), amplification of the *SMN1* gene was absent (Table 1 and Figure 2), whilst the TRECs and KRECs values were normal (TRECs = 107 and 45 and KRECs = 47 and 29 copies/punch, respectively). The newborns were transferred to the Paediatric Neurology Unit of the Hospital Virgen del Rocio to undergo clinical and genetic evaluation. Case A showed respiratory distress and hypotonia. The genetic results (MLPA) of Sample A (47 days-old), showed absence of the *SMN1* gene and 2 copies of the *SMN2* gene. The patient was diagnosed with SMA type 1 and treatment with intrathecal Nursinensen was started at 60 days of life. Currently, this child continues to suffer from marked respiratory, oropharyngeal and gastrointestinal symptoms. Case B (28 days old) presented with severe SMA respiratory and neuromotor symptoms at birth. The sample B also showed absence of the *SMN1* gene and 2 copies of the *SMN2* gene (MLPA) and was diagnosed with SMA type 1; sadly, his disease was rapidly progressive and the patient died prior to initiation of specific treatment with intrathecal Nursinensen.

**Figure 3:**
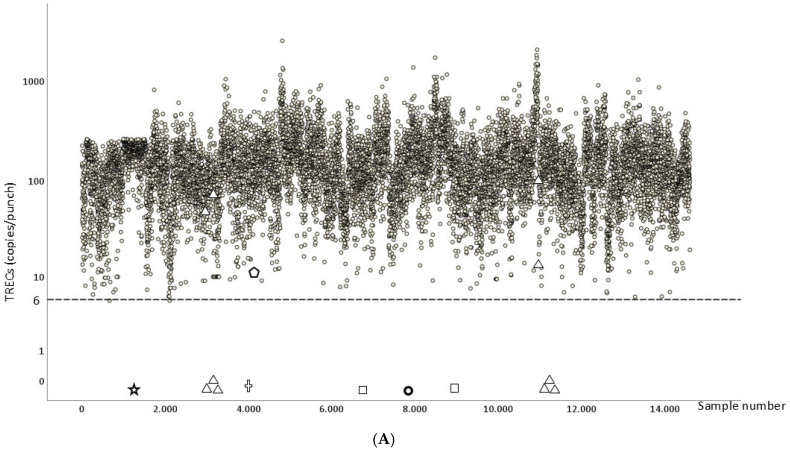

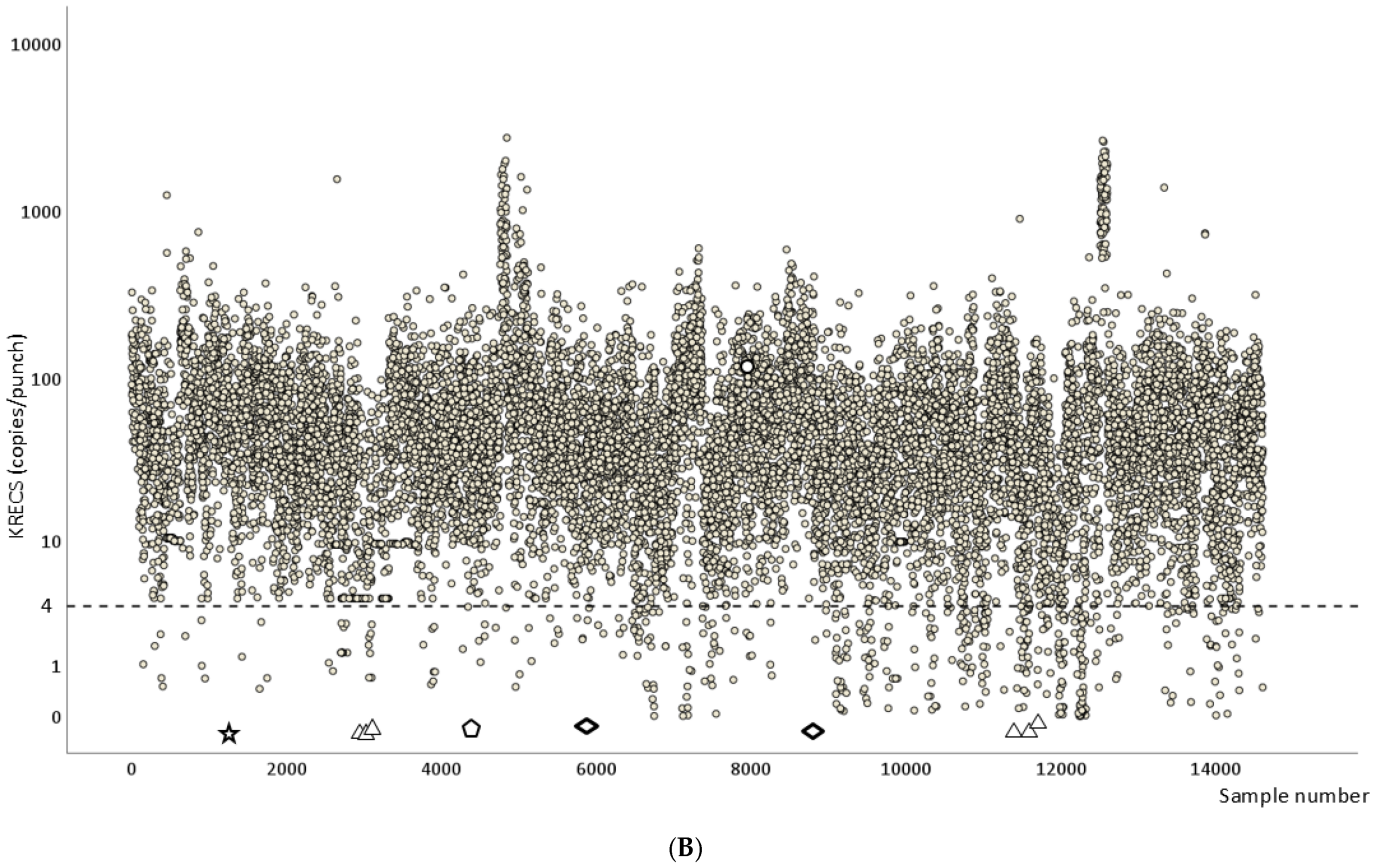
(**A**): TRECs values from DBS and controls: 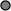 prospectively enrolled newborns; 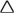 CDC controls; 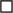 IL7-R SCID controls; 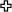 IL2RG Control; 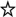 SCID Suspected (case C); 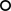 SCID Suspected (case D); 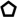 XLA Suspected (case E); - - - grey dotted line: cut-off for TRECs<6 copies/punch. (**B**) KRECS values from DBS and controls grey: 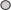 prospectively enrolled newborns;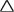 CDC controls; 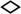 XLA control; 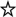 SCID Suspected (case C); 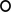 SCID Suspected (case D); 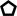 XLA Suspected (case E); - - - grey dotted line: cut-off for KRECs<4 copies/punch.

Figure 3A,B summarizes the results of the TRECs and KRECs/punch; no severe T- and/or B-cell lymphopenia were detected prospectively. In the results obtained for TRECs (Figure 3A), the median of the TRECs obtained was 132 [79–208] copies/punch (median [interquartile range]) and similar to those obtained in previous studies [30]. In 48 (0.34%) samples it was necessary to repeat the technique on the same DBS sample (re-test) due to values below the cutoff points. and of these, on 21 occasions, a second dried blood sample was required (re-sample) due to pathological or inconclusive results for TRECs in 21 (0.15%) DBS samples. We included ten (six positive and four negative) CDC samples, two internal IL7-R SCID, and one internal IL2Rγ-SCID, and all of them were correctly detected, showing the pathological TRECs levels. During the study, the DBSs of two newborns (cases C and D) with suspected PID (primary immune deficiency) were included retrospectively. Case C involved a 3-month-old infant (sample: Table 1, Figure 3A,B) whose DBS was stored in the metabolic laboratory of our centre and showed pathological values for TRECs and KRECs (0 copies/punch each), whilst *SMN1* amplified correctly (Ct = 30). Subsequently, flow cytometry showed an absence of T, B and NK cells (T^−^B^−^NK^−^) and NGS showed that the patient had pathogenic alterations in heterozygosis in the *DCLRE1C* and *ELANE* genes (c.95C>T/c.217G>A) and also showed a heterozygous deletion of 60 KB affecting the *DCLRE1CP1* and *MEIG1* genes and the first three exons of the *DCLRE1* gene. Genetic alterations in heterozygosis of the *DCLRE1C* gene ((c.95C>T) mutation and gross deletion) are clinically associated with Artemis-SCID [32,33]. Supportive therapy was initiated and curative treatment in the form of gene therapy was successfully performed. 

Newborn DBS from a then 5-month-old infant with suspected IEI due to severe hypogammaglobulinemia (case D, Table 1, Figure 3A,B) showed pathological values for TRECs and normal values for KRECs (0 and 117 copies/punch, respectively), whilst *SMN1* amplification was normal (Ct = 29). In this case, flow cytometry showed an absence of T cells (T^−^B^+^NK^+^) and the patient’s NGS study showed a homozygous pathogenic variant in the *ATM* gene (c.8585-2A>C) associated with ataxia telangiectasia (AT) [32]. This patient subsequently started on immunoglobulin replacement therapy and prophylaxis while awaiting inclusion in a clinical trial for the management of children with AT. 

In addition, we also tested a DBS sample retrospectively from a 6-month-old infant with suspected XLA (Sample E, Table 1). We confirmed normal TRECs values (18 copies/punch) and absent KRECs (0 copies/punch) with normal *SMN1* amplification (Ct = 29). Absence of BTK protein expression confirmed X-linked agammaglobulinemia (XLA).

The results obtained in relation to the KRECs were very variable (Figure 3B); the median of KRECs obtained was 40 [17–77] copies/punch (median [interquartile range]). Re-test was necessary in 138 (0.98%) samples due to values below the cutoff points, and of these, re-sample of a second DBS was required in 124 (O.88%) due to pathological or indeterminate results for KRECs. CDC and internal controls (XLA) were all included and correctly identified (Figure 2). After the acquisition of the second batch of reagents, the repetition rate increased markedly to 3.6%. In this way, we were able to observe how three or four samples of each reaction (plate) showed false pathological levels below the cutoff points, thus increasing the number of re-tests. After evaluating and consulting with the reagent manufacturer (LightMix^®^ TREC KREC SMA Newborn Screening Kit, Roche Diagnostics, Berlin, Germany), we concluded that these reagents did not meet our laboratory’s minimum quality standards. As a result, we are considering other commercial companies for future studies.

## 4. Discussion

Here we report for the first time the results of a prospective simultaneous neonatal screening for SMA and severe T- and B-cell lymphopenia in the Spanish population. A total of 35,000 children were born in Western Andalusia in 2020. Considering that the estimated incidence rates of SMA and SCID are 1/6000–1/10,000 and 1/54,000–1/10,000, respectively [34,35,36,37], we expected to detect two to three infants with SMA and zero or one severe T- and B-cell lymphopenia case. However, no cases of SMA or STBCL were detected. This is likely due to the fact that not all neonates in our region were prospectively included as part of this pilot study. However, two infants with SMA and one each with Artemis-SCID, AT and XLA were identified retrospectively using the applied technique. The pathological results supported the clinical presentation and were confirmed by phenotypic and genotypic studies.

Newborn blood screening [38] using DBS is performed on babies at birth to detect disorders that cause life-threatening intellectual or physical illnesses [39]. The number of pathologies screened depends on the country and region. Furthermore, the improvement of DNA-based technologies has changed the NBS model to allow multiple screenings for several disorders simultaneously as SMA or SCID. The inclusion of these pathologies in the NBS started in 2008 with severe T-cell lymphopenias in Wisconsin (USA) [16] and has since been implemented in several countries and regions including Israel, New Zealand, Norway, Taiwan, several provinces in Canada, Switzerland, Germany, Iceland, Sweden, Italy (Tuscany), Spain (Catalonia) and in some regions in Australia [38,40,41]. The inclusion of severe T-cell lymphopenias in routine neonatal screening allows for prompt supportive care followed by curative management, resulting in an increased overall survival whilst potentially being cost-effective [18,42,43]. The combined TREC/KREC assay has some advantages such as the identification of patients with late-onset ADA, X-linked agammaglobulinemia, autosomal recessive agammaglobulinemia or cases of Nijmegen breakage syndrome [44]. The early detection and treatment of these pathologies also reduces their morbidity and mortality [45]. However, the inclusion of KRECs in the NBS program is controversial. Several studies have demonstrated low sensitivity expressed by a high rate of false positive results associated with additional costs [30,44,46,47]. Indeed, we have also observed a high variability and a high false positive rate in the determination of KRECs, up to (3.6%), posing an increased level of unnecessary distress and anxiety for the infant and the family [26,28]. During our study, we have observed that the quality of the KREC determination reagents has been inconsistent. In addition, false positives can be due to low gestational age and birth weight as well as the use of immunomodulatory therapy during pregnancy [30,48]. It was not possible to collect individual demographic data (such as gestational age or birth weight) in our cohort. However, we estimate that 15–20% of newborns in our region were born preterm and/or had low birth weight. We observed an increase in the false-positive rate of KRECs in our study, particularly following the acquisition of the second batch of reagents. Therefore, we conclude that the kit used in this study does not meet the reliability requirements necessary for accurate quantification of KRECs. At the moment of the study, there were few companies that provided reagents for the joint determination of TRECs/KRECs and *SMN1* with the CE-IVD classification (European in vitro diagnostic medical device). In the case of our study, we opted for the reagents we had tested previously (although not including *SMN1*) [30]. It would be worth exploring other reagents that are currently available and on which research has been published [27,49].

The results in respect to the TRECs were similar to those obtained in our previous work [30]. The technique allows the diagnosis of suspected cases of PIDD quickly, allowing prompt initiation of supportive and curative treatment. The early diagnosis of these pathologies is very important and will determine disease progression and the overall outcome. As mentioned above, survival is reported to exceed 90% in infants diagnosed with SCID undergoing curative therapy in the form of HSCT or gene therapy (GT) early in life and in the absence of infection [18].

The inclusion of SMA does not pose a major difficulty nor interfere with the TRECs and KRECs results. We observed reassuring homogeneity of the results obtained; nearly all of the samples amplified the *SMN1* gene in the same cycle, whilst control cases and cases suspected of SMA were correctly identified as pathological. Early diagnosis is extremely important because a rapid loss of motor neurons occurs long before the onset of symptoms [9] and because the curative treatment should be initiated prior to the onset of symptoms in order to obtain the optimal therapeutic effects [38]. Countries such as Belgium are currently including SMA in the NBS program [50] after being supported by several cost-effectiveness studies [28,51,52]. Finally, there are few studies on the joint detection of SCID and SMA. Importantly, a study from Australia suggests combined NBS for severe lymphopenias and SMA to be cost effective [28].

In this paper, we present the results of the first prospective study of neonatal screening for SMA and STBCL in Western Andalusia. The results obtained demonstrated the applied technique and subsequent circuit involving clinicians and laboratory specialists to be reliable and rapid for the detection of SMA and SCID from DBS. Our centre is constituted by healthcare professionals from all areas involved in the diagnosis and treatment of SMA and severe T- and/or B-cell lymphopenias (paediatricians, neuro-paediatricians, geneticists and immunologists). During this pilot study, we received DBSs from suspected cases (SMA or STBCL), all of which were correctly identified or excluded, and according to the protocol, rapidly referred to the clinical and laboratory specialist to confirm the diagnosis followed by appropriate treatment initiation. Sadly, treatment of symptomatic infants diagnosed with SMA did not result in reversal of the earlier manifestations, leaving the affected infant with important sequelae, further highlighting the need of diagnosis prior to suffering from SMA-related clinical symptoms.

A major limitation of this pilot project was the inability to screen the entire newborn population prospectively during the study period. However, it is promising that the Spanish Ministry of Health has recently committed to including SCID and SMA in the NBS program in 2025, which will allow early diagnosis and initiation of supportive and curative treatment of affected newborns and infants, thus improving the quality of life of affected patients and their families.

## Figures and Tables

**Table 1:** Results obtained in suspected cases.

Case/ Sample	*SMN1* (Ct)	TRECs (copies/punch)	KRECs (copies/punh)	Genetic Study **	Final Diagnosis
A	NA *	107	47	Absence *SMN1* 2 copies *SMN2*	SMA 1B
B	NA *	45	29	Absence *SMN1* 2 copies *SMN2*	SMA 1B
C	30	0	0	c.95C>T/c.217G>A (*DCLRE1CP1/MEIG1* genes) and (60 Kb deletion 3 first exons of *DCLRE1* gene)	Artemis-SCID (T^−^K^−^NK^−^)
D	29	0	117	C8585-2A>C (*ATM* gene)	ATM (T^−^B^+^NK^+^)
E	29	18	0	-	XLA (T^+^B^−^)

* NA not amplify, positive for SMA; ** Genetic study: *SMN1* by MLPA (Multiplex Ligation-dependent Probe Amplification) and PID by NGS (Next Generation Sequencing).

## Data Availability

The data that support the findings of this study are available from the corresponding author upon reasonable request.
